# Evaluating Cognitive Screening Tools for Older Adults in Ambulatory Care Settings

**DOI:** 10.1093/geroni/igaf122.3161

**Published:** 2025-12-31

**Authors:** Shannon Foran, Grace Armstrong, Sherry Greenberg, Nicholas Schiltz, Anne Pohnert, Mary Dolansky

**Affiliations:** Case Western Reserve University, Frances Payne Bolton School of Nursing, Cleveland, Ohio, United States; Case Western Reserve University, Cleveland, Ohio, United States; Hunter-Bellevue School of Nursing, New York, New York, United States; Case Western Reserve University, Cleveland, Ohio, United States; CVS Health, Woonsocket, Rhode Island, United States; Case Western Reserve University, Cleveland, Ohio, United States

## Abstract

Implementation of the Age-Friendly Health Systems 4Ms framework ensures that assessing and acting on What Matters, Medication, Mentation, and Mobility is included for all patients aged 65 and older. In ambulatory settings, patients in this age group are often assessed annually for dementia using brief cognitive screening tools to screen for Alzheimer’s Disease/Related Dementias (ADRD). Providers often report that older adults are hesitant to engage in the screening, frequently stating that they do not perceive any memory issues. Additionally, controversy exists on the benefits for screening all older adults. The purpose of this literature review is to present: (a) current instruments used for cognitive screening such as the Mini-Cog, the Eight-item Informant Interview to Differentiate Aging and Dementia (AD8®), Memory Impairment Screen (MIS), and Montreal Cognitive Assessment (MoCA); (b) barriers to dementia screening from both provider’s and patients’ perspectives; and (c) the ongoing controversy surrounding population screening for dementia in the ambulatory care setting. This review will include a comparison of these instruments in terms of time to complete, acceptability, specificity, and sensitivity. Current utilization and recommendations for these screening tools in ambulatory care settings will be reported. Strategies for implementing these assessments in ambulatory care, where patients often expect short and succinct appointments, will also be presented.

